# Foodborne Pathogen Campylobacter

**DOI:** 10.3390/microorganisms9061241

**Published:** 2021-06-08

**Authors:** Nicolae Corcionivoschi, Ozan Gundogdu

**Affiliations:** 1 Bacteriology Branch, Veterinary Sciences Division, Agri-Food and Biosciences Institute, Newforge Lane, Belfast BT9 5GB, UK; nicolae.corcionivoschi@afbini.gov.uk; 2 Department of Infection Biology, Faculty of Infectious & Tropical Diseases, London School of Hygiene & Tropical Medicine, Keppel Street, London WC1E 7HT, UK

*Campylobacter* is the most common bacterial cause of human gastroenteritis in the world, with the species *Campylobacter jejuni* being responsible for over 80% of *Campylobacter* infections [1]. *C. jejuni* is abundant within the avian gut and the consumption and handling of poultry is the main route of transmission to humans [2,3]. In humans, *C. jejuni* infection ranges from asymptomatic carriage to bloody diarrhoea, fever and abdominal pains as well as serious post-infectious sequelae such as the neuromuscular paralysis of Guillain–Barré syndrome [4]. In low-resource areas, *Campylobacter* infections are common in young children (causing watery diarrhoea rather than the bloody diarrhoea that occurs in high-resource countries) and are associated with many deaths, as well as stunted growth and life-long physical and cognitive deficiencies [5]. In addition, as highlighted by the WHO, *C. jejuni* is a multi-antibiotic-resistant pathogen and new therapeutics are urgently required [6].

Historically, the lack of a convenient animal model, coupled with genetic diversity and difficulties in culturing *C. jejuni*, has hampered pathogenesis research [7]. For this reason, it is important that the Campylobacter research community continues to investigate this important human pathogen. This Special Issue was produced with the aim of promoting the latest research on *Campylobacter* pathogenicity focusing on a range of topics from virulence determinants such as lipooligosaccharide (LOS) to functional characterisation of specific genes of interest. The Special Issue aims to look at novel infection models, immunological responses of *Campylobacter* infection and epidemiological studies with the overarching aim of intervention and control strategies. Survival themes such as biofilms, antimicrobial resistance and omics-based topics such as the microbiome also form a key part of the Special Issue. Overall, this *Microorganisms* Special Issue highlights some of the most up-to-date research relating to *Campylobacter* pathogenicity.

Our aim to further understand the pathogenicity and physiology of *Campylobacter* has been pursued by a number of research groups. Kovács et al. [8] investigate the virulence traits of inpatient *C. jejuni* isolates and apply a transcriptomic approach to identify potential genes maintaining a role in intracellular survival. Characteristic groups of genes were identified as significantly upregulated, outlining a survival strategy of internalised *C. jejuni*, comprising genes related (1) to oxidative stress; (2) to a protective sheath formed by the capsule, LOS, N-, and O-glycosylation systems; (3) to dynamic metabolic activity supported by different translocases and the membrane-integrated component of the flagellar apparatus; and (4) to hitherto unknown genes. Talukdar et al. [9] investigate *C. jejuni* CadF and FlpA virulence proteins in binding to host cell fibronectin. The authors demonstrate that the *C. jejuni* CadF and FlpA adhesins facilitate the binding of *C. jejuni* to the host cells, permit delivery of effector proteins into the cytosol of a host target cell and aid in the rewiring of host cell signalling pathways to alter host cell behaviour. Guirado et al. [10] discuss the differential distribution of the *wlaN* and *cgtB* genes, which are associated with Guillain–Barré Syndrome in *C. jejuni* isolates from humans, broiler chickens and wild birds. The authors detect two variants of a G-rich region within the *cgtB* gene, suggesting that, similarly to *wlaN*, the G-tract in the *cgtB* gene mediates the phase variation control of *cgtB* expression. Guk et al. [11] investigate hyper-aerotolerant *C. coli* from duck sources and their increased potential of virulence, antimicrobial resistance and genetic relatedness. Recently, aerotolerant (AT) *C. jejuni* with the ability to survive under aerobic stress has been reported. The authors investigate the prevalence of hyper-aerotolerant (HAT) *C. coli* from duck sources with these strains most likely being transmitted to humans through the food chain given their aerotolerance.

A number of research articles investigate infection models and/or immunological aspects of *C. jejuni* infection. Kløve et al. [12] show that the immunopathological sequelae in *C. jejuni*-infected mice were due to Toll-like receptor 4 (TLR4)-dependent immune responses induced by bacterial LOS. Here, the authors further investigate and identify TLR4 involved in mediating *C. coli* LOS-induced immune responses in intestinal and extra-intestinal compartments during murine campylobacteriosis. Heimesaat et al. [13] investigate the use of the polyphenolic compound resveratrol to alleviate acute *C. jejuni*-induced enterocolitis in a preclinical intervention study. Functional analyses revealed that resveratrol treatment could effectively rescue colonic epithelial barrier function in *C. jejuni*-infected mice. The authors describe that peroral resveratrol treatment does exert potent disease-alleviating effects during acute experimental campylobacteriosis. Heimesaat et al. [14] also investigate the immune-modulatory properties of the octapeptide NAP in *C. jejuni*-infected mice suffering from acute enterocolitis. The authors discuss how they used an acute *C. jejuni* induced enterocolitis model, and they surveyed the anti-pathogenic and immune-modulatory effects of the octapeptide NAP, which is well-known for its neuroprotective and anti-inflammatory properties. NAP treatment resulted in less distinct innate and adaptive pro-inflammatory immune responses that were not restricted to the intestinal tract but could also be observed in extra-intestinal and even systemic compartments. NAP treatment further resulted in less frequent translocation of viable pathogens from the intestinal tract to extra-intestinal areas including systemic tissue sites.

In conjunction with basic laboratory research, applied research is important for us to investigate *Campylobacter* in the real world. Rapp et al. [15] study the importance of the farm environment and wildlife for transmission of *C. jejuni*, specifically in a pasture-based dairy herd. The results indicate that management of grazed pasture and supplementary feed contaminated by bird droppings could be targeted to effectively reduce transmission of *C. jejuni* to dairy herds, the farm environment and, ultimately, to humans. Šimunović et al. [16] compare *C. jejuni* from slaughterhouse and surface-water isolates, indicating the better adaptation of the former to the chicken host environment. The authors highlight adaptation of *C. jejuni* slaughterhouse isolates to the chicken host, as well as increased biofilm cell resistance due to increased efflux pump activity. Di Donato et al. [17] investigate the prevalence, population diversity and antimicrobial resistance of *C. coli* isolated in Italian swine at slaughterhouses. The authors identify a strong correlation between phenotypic and genotypic resistance to fluoroquinolone and tetracycline. Liang et al. [18] investigate the development of a lyophilisation process for *Campylobacter* bacteriophage storage and transport. In this study, the authors describe the development of a lyophilisation approach to maintain phage titers, ensure efficacy and reduce transport costs of *Campylobacter* bacteriophages.

As with all modern science, the use of in silico bioinformatics approaches to study relevant research questions goes hand in hand with laboratory science. Bandoy and Weimer [19] use machine learning combined with *Campylobacter* population genomics to reveal virulence gene allelic variants that cause disease. The authors validated a novel framework to define infection mechanism using a combination of a GWAS, machine learning and bacterial population genomics that ranked allelic variants to identify disease.

The Special Issue also provides three reviews. Pumtang-on et al. [20] perform a systematic review of *C. jejuni* vaccine candidates for chickens, highlighting the need for increased consistency in the way *C. jejuni* vaccine studies in poultry are designed and reported in order to be able to undertake a robust comparison of *C. jejuni* vaccine candidates. Mousavi et al. [21] discuss a novel clinical *C. jejuni* infection model based on sensitisation of mice to LOS. The review highlights the major role of LOS-driven innate immunity in pathogenesis of campylobacteriosis, including post-infectious autoimmune diseases, and promote the preclinical evaluation of novel pharmaceutical strategies for prophylaxis and treatment. Tram et al. [22] discuss *C. jejuni* and biofilms. This review examines factors that can trigger biofilm formation and is a timely reminder of the importance of biofilms in relation to *C. jejuni* survival.

This Special Issue highlights the latest developments within *Campylobacter* research, particularly those relating to pathogenicity.
